# Sexual Signalling in *Propithecus verreauxi:* Male “Chest Badge” and Female Mate Choice

**DOI:** 10.1371/journal.pone.0037332

**Published:** 2012-05-17

**Authors:** Stefania Dall'Olio, Ivan Norscia, Daniela Antonacci, Elisabetta Palagi

**Affiliations:** 1 Centro Interdipartimentale Museo di Storia Naturale e del Territorio, Università di Pisa, Pisa, Italia; 2 Dipartimento di Biologia Evoluzionistica, Università di Firenze, Firenze, Italia; 3 Istituto di Scienze e Tecnologie della Cognizione , Consiglio Nazionale Delle Ricerche, Roma, Italia; University of California Santa Barbara, United States of America

## Abstract

Communication, an essential prerequisite for sociality, involves the transmission of signals. A signal can be defined as any action or trait produced by one animal, the sender, that produces a change in the behaviour of another animal, the receiver. Secondary sexual signals are often used for mate choice because they may inform on a potential partner's quality. Verreaux's sifaka (*Propithecus verreauxi*) is characterized by the presence of two different morphs of males (bimorphism), which can show either a stained or clean chest. The chest becomes stained by secretions of the sternal gland during throat marking (rubbing throat and chest on a vertical substrate while smearing the scent deposition). The role of the chest staining in guiding female mate choice was previously hypothesized but never demonstrated probably due to the difficulty of observing sifaka copulations in the wild. Here we report that stained-chested males had a higher throat marking activity than clean-chested males during the mating season, but not during the birth season. We found that females copulated more frequently with stained-chested males than the clean-chested males. Finally, in agreement with the biological market theory, we found that clean-chested males, with a lower scent-releasing potential, offered more grooming to females. This “grooming for sex” tactic was not completely unsuccessful; in fact, half of the clean-chested males copulated with females, even though at low frequency. In conclusion, the chest stain, possibly correlated with different cues targeted by females, could be one of the parameters which help females in selecting mates.

## Introduction

Communication, an essential prerequisite for sociality, involves the transmission of signals [1], [2]. A signal can be defined as any action or trait produced by one animal, the sender, that produces a change in the behaviour of another animal, the receiver [3]. The transfer of messages, either born or not by signals [4], can be beneficial to either senders, receivers, or both [5]. Secondary sexual signals (visual, acoustic or chemical) are often used for mate choice because they may inform on a potential partner's quality [6]. In bipedal vertebrates, mate choice often relies on visual sexual signals that are placed frontally to the observer. This situation occurs quite frequently in birds. Peacock (*Pavo cristatus*) tail spreading [7], the level of symmetry in chest plumage of male zebra finches (*Taeniopygia guttata*) [8], and the size of the black feather bib on the throat of male house sparrows (*Passer domesticus*) [9] are just three examples of secondary visual sexual signals used by females to choose mating partners. Within the primate order, some observers have reported that also in humans (*Homo sapiens*), women's sexual selection appears to be influenced by the amount of chest hairs in males [10]. Men's choice can be affected by size and symmetry of women's breasts [11]–[13], a signal that in humans is exaggerated compared to other primates [14], [15]. Quadruped locomotion habits and chest sexual signals do not generally co-exist due to obvious perceptual constraints.

Frontal visual signals can be favoured by sexual selection when three conditions are met: 1) a diurnal lifestyle, which makes visual signals detectable; 2) upright locomotion, which makes face and/or chest signals visible; 3) a mating system based on either female or male mate choice and strong intra-sexual competition [16].

In primates, besides humans, only a few species meet such conditions [17]. Orang-utans are one of the most sexually dimorphic apes with dimorphism in size, adornments, and vocal signals [17]. Orang-utans are characterized by an irreversible bimorphism and fully mature males can show frontal sexual adornments, which consist of cheek flanges and a throat pouch, a sort of chest “badge” [18]. Males without such secondary sexual features are generally named as “unflanged” males which, under particular social circumstances (e.g. the absence of a flanged male), can acquire in a few months the adornments typical of flanged males [19].

**Figure 1:**
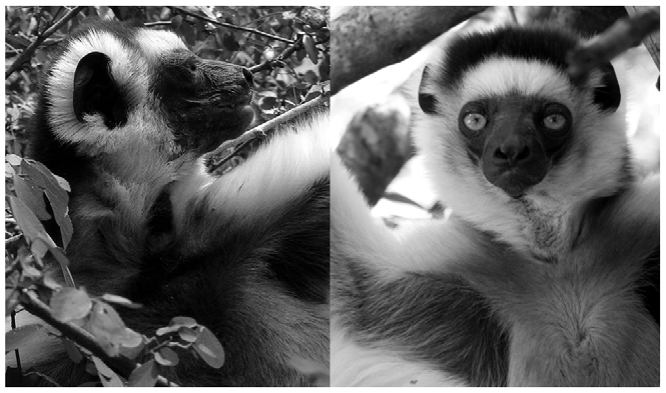
The two different morphs of sifaka males. An example of stained-chested male (on the left, photo by E. Palagi). The brown smear, particularly evident on the throat, extends to the upper part of the chest (dark/brown, photo by I. Norscia). An example of clean-chested male (on the right). No brown smear is present on the throat.

Verreaux's sifaka (*Propithecus verreauxi*) is a diurnal prosimian species characterized by upright locomotion (bipedal hopping and leaping; [20]) and male intra-sexual competition [21]. There is a lack of sexual dimorphism in body size and females are dominant over males [20], [22]. These characteristics make it impossible for males to coerce female copulation and promotes female mate choice [20], [22]–[24]. Lewis [25] reported bimorphism in male sifakas, which can show either a stained or unmarked chest, as a function of scent-marking activity during which the throat and chest are rubbed up against a substrate, often multiple times within a single marking bout [26]; Table 1. (Fig. 1). In prosimians, glandular scent-marking has a variety of social functions such as advertisement and territorial defense (*Propithecus verreauxi*, [25]; *Propithecus edwardsi*, [27]; *Lemur catta* [28]), intergroup communication (*Propithecus verreauxi* [25]), advertisement of social dominance (*Lemur catta*, [29]), signaling of reproductive condition (*Lemur catta*, [30], [31]), and mate selection (*Nycticebus pygmaeus* [32]; *Propithecus verreauxi*, [22]). About half of the scent marks by sifaka males are overmarks, in which a scent mark is placed on or near a female scent mark [25] and thus, in cases of intense activity, the staining of the chest is probably a combination of a male's own glandular secretions, female anogenital secretions, female urine, and dirt [26]. Lewis and van Schaik [26] described this male phenotypic variation (stained versus clean-chested males) as a form of reversible bimorphism. However, the authors did not find any clear evidence that the two morphs of males differ in their intrinsic physical characteristics, such as body size and maxillary canine length [26]. Norscia et al. [22] demonstrated that females gave copulatory priority to males who more frequently countermarked female scent depositions. However, results demonstrating a clear link between male chest bimorphism and female mate choice are still lacking.

**Table 1:** Description of the behaviours recorded during the study.

Behavioural items	Description
Mating event	Copulatory behaviour in which intromission and thrusting are unambiguously observed. Ejaculation, generally not visible, is inferred based on a rapid increase in thrusts and a pause just prior to the dismount, followed by intense genital self-grooming [45], [55].
Grooming	Fur-cleaning, which in strepsirhines is typically performed via tooth-comb.
Genital marking	The genitals are rubbed on the substrate and scent deposition is released. Both males and females perform genital marking.
Throat marking	Animals rub their throat and chest on a vertical substrate in a repeated manner while smearing the scent deposition. Throat marking is a dimorphic behaviour, in fact only males perform it.

Here, we decided to test whether or not sifaka females' mating patterns are associated with the male chest badge, which seems to correlate with male scent-marking and dominance [25], [26]. We made the following predictions:

### Prediction 1

Lewis's findings [25] suggest that while clean-chested males deposit scents for inter-group communication, stained-chested males release scent depositions for mate-guarding purposes. Moreover, during the birth season, testes mass (and, possibly, in testosterone levels) do not differ between clean- and stained-chested males [33]. If the stained chest is a signal linked to male intra-sexual competition (ultimate cause) and to testosterone levels (proximate cause), we expect stained-chested males to show a higher throat-marking frequency than the clean-chested males during the mating season but not necessarily during the birth season.

### Prediction 2

Lewis and van Schaik [26] reported that stained-chested males are generally dominant in their social groups. Thus, if a stained chest is one of the possible signals which females can use in their mate choice, we predict stained-chested males will copulate more frequently than the clean-chested males.

### Prediction 3

In the mating market, the balance of power tilts in favor of females whenever males cannot force them into mating [24], especially when females are dominant. Consequently, males depend on females for breeding opportunities and must compete to prove their superiority to females, thus increasing their possibility to be selected [24], [34]. Males can engage in both contest competition via physical/ritualized fighting and outbidding competition, in which a male plays off rivals by making a better offer [35]. In the latter case, males can secure the favors of a female by advertising their quality (e.g. the dominance status) through visual or olfactory displays [36], [37] and/or by providing commodities in exchange for female access [38], [39]. In sifaka, the mating system follows the biological market rules where both scent-marking and grooming are good male services on which females base their mate selection [22]. Norscia et al. [22] found that to obtain priority and/or a high number of copulations sifaka males had to be top-scent releasers and/or females' top-groomers. According to the biological market theory, we expect that clean-chested males, with lower scent-releasing potential [26], in order to have some copulation opportunities need to compensate by offering more grooming to females than stained-chested males.

## Results

### Prediction 1 supported

**Figure 2:**
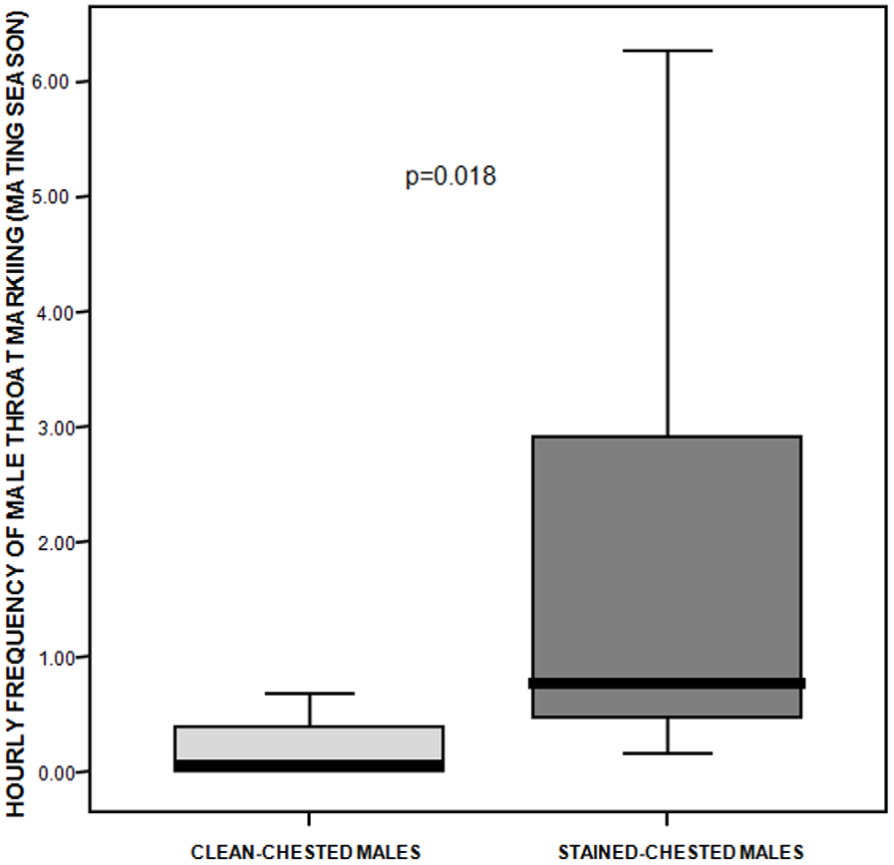
Marking activity in the mating season. Frequency of throat marking performed by clean- and stained-chested males during the mating season. Solid horizontal lines indicate medians; length of the boxes corresponds to inter-quartile range; thin horizontal lines indicate range of observed values.

During the mating season stained-chested males (mean ±SE: 2.07±1.15 times per hour) throat marked significantly more often than clean-chested males (mean ±SE: 0.19±0.11 times per hour) (two independent samples randomization test: t = −1.789; n_c_ = 6, n_s_ = 5, p = 0.018; Fig. 2). A significant difference was also found in the use of genital glands by the two morphs of males (stained-chested males, mean ±SE: 0.29±0.17 times per hour; clean-chested ones, mean ±SE: 0.02±0.01 times per hour; two independent samples randomization test: t = −1.688, n_c_ = 6, n_s_ = 5, p = 0.045).

During the birth season the difference in the throat-marking between stained- and clean-chested males disappeared (stained-chested males, mean ±SE: 3.52±1.03 times per hour; clean-chested ones, mean ±SE: 0.54±0.26 times per hour; two independent samples randomization test: t = 2.801, n_c_ = 3, n_s_ = 3, p = 0.140); no difference was also found for genital depositions (stained-chested males, mean ±SE: 1.49±0.19 times per hour; clean-chested ones, mean ±SE: 0.07±0.18 times per hour; two independent samples randomization test: t = 7.151, n_c_ = 3, n_s_ = 3, p = 0.105). The seasonal difference in the sample size (11 males, mating season; 6 males, birth season) is due to the presence of out-group males in our study groups during the mating period [40].

### Prediction 2 supported

**Figure 3:**
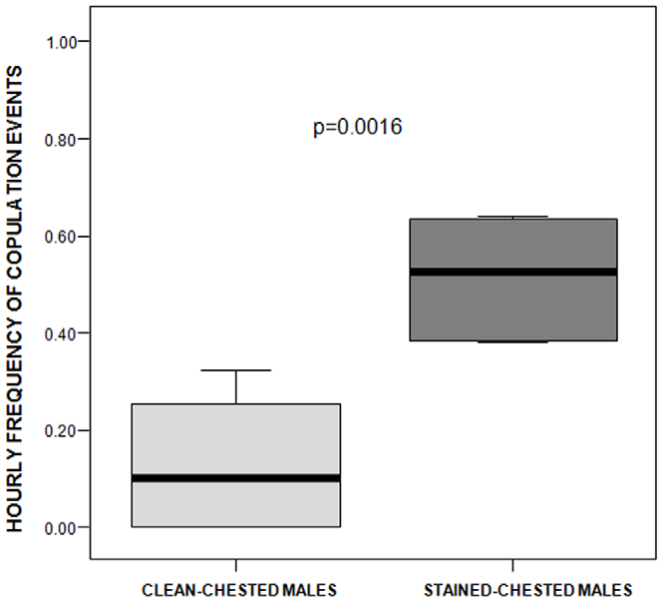
Copulation events of the two different morphs of males. Frequency of copulation events performed by clean- and stained-chested males. Solid horizontal lines indicate medians; length of the boxes corresponds to inter-quartile range; thin horizontal lines indicate range of observed values.

In the mating season, the stained-chested males engaged in significantly more copulation events per hour (mean ±SE: 0.59±0.12) than the clean-chested ones (mean ±SE: 0.12±0.06) (two independent samples randomization test: t = −3.587, n_c_ = 6, n_s_ = 5, p = 0.0016; Fig. 3).

### Prediction 3 supported

**Figure 4:**
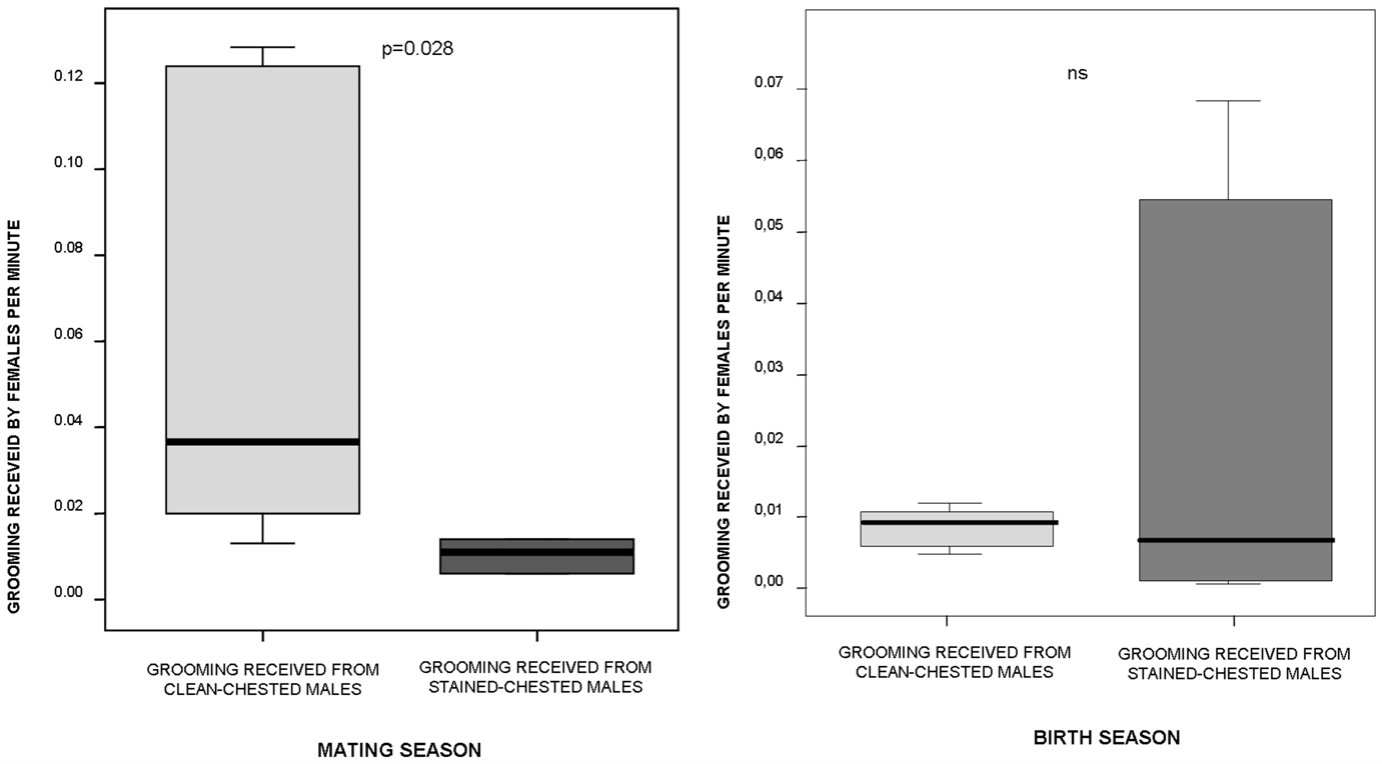
Grooming received by females. Frequency of grooming received by each female from clean- and stained-chested males (a) during the mating season and (b) during the birth season. Solid horizontal lines indicate medians; length of the boxes corresponds to inter-quartile range; thin horizontal lines indicate range of observed values.

In the mating season females received more grooming from clean-chested males (mean ±SE: 0.06±0.02 times per minute) than from stained-chested ones (mean ±SE: 0.14±0.00 times per minute) (paired samples randomization test: t = 2.035, n = 6, p = 0.028; Fig. 4a). This difference vanished in the birth season (paired samples randomization test: t = −0.81, n = 6, p = 0.499; Fig. 4b).

## Discussion

In this paper, we found that stained-chested males had a higher throat and genital-marking activity than clean-chested males during the mating season but not during the birth season (Prediction 1 supported). Moreover, we found that females copulated more frequently with stained-chested males (including both resident and non-resident) than with clean-chested males (including both resident and non-resident) (Prediction 2 supported), even though the latter offered more grooming to females compared to the former during the mating season (Prediction 3 supported).

Males of several mammalian species modulate their scent-marking activity according to their perceived mating opportunities [41], [42] and can increase their plasma testosterone concentration, as well as scent-marking, when they are sexually stimulated [43]. In the mating season a scent-marking dichotomy between the two different morphs of sifaka males existed (stained-chested males scent-marked more frequently than clean-chested ones). This dichotomy disappeared during the birth season, when males were not sexually stimulated and males' intra-sexual competition decreased due to the lack of eggs to be fertilized. One of the proximate causes of the scent-marking dichotomy in the mating season is the difference in the concentration of testosterone levels between stained- and clean-chested males, which also differ in their testes mass [33]. The lack of difference in the testes mass of the two male morphs during the birth season led authors [33] to infer that stained- and clean-chested males do not differ in their testosterone levels. This is consistent with our data, which show no difference in the frequency of scent-marking rates between the two morphs of males in the birth season.

The stained-chest provides benefits to sifaka males by increasing their reproductive opportunities (“marking for sex” tactic). Copulations involved both in-group and out-group stained-chested males, this suggests that the chest badge can be functional to females, especially when they have to gather information on less familiar out-group males. This interpretation is supported by another recent finding obtained from Beza Mahafaly sifaka population, where it has been observed that most (29 of 52) of males sired at least one offspring outside their resident group [44].

Lewis and van Schaik [26] underlined the importance of multimodal signaling in *Propithecus verreauxi*, in which the additional visual cue of a chest stain enhances the information transmitted via the olfactory signal produced by the scent glands. Signals are frequently made up of multiple components that interact with each other to alter the receiver's response [4], [44]–[46]. Such multiple signals were defined as multimodal (composed of signals related to different sensory modalities) [47], [48]. The multimodality of sifaka communication is linked to its diurnal habits [20]. In fact, diurnal prosimians use multimodal signals in both reproductive and nonreproductive contexts [26], [49], [50]. Two different studies showed that both *Propithecus edwardsi* and *Microcebus murinus* females use multimodal estrus advertisement by associating a particular vaginal morphology with vocalizations [51], [52]. Palagi et al. [49] and Palagi and Dapporto [53] described urine-marking in *Lemur catta* as a multimodal signal composed by an olfactory cue (urine) and a visual cue (tail up, increasing the detection probability). Switching from unimodal (one cue) to multimodal signalling (more than one cue) may increase the probability of sifaka males to be promptly detected by females. Sifaka males adjust the intensity of their signal by varying its delivery frequency. Maintaining the visual chest badge is likely to be costly because it requires much effort in renewing scent depositions.

In many non-human primate species, grooming is a commodity which can be exchanged for itself or for breeding opportunities [54]–[56]. Grooming is one of the behaviors most frequently involved in the biological market system [35]. Within a mating marketplace, low quality males are expected to overcompensate for their quality by providing more grooming to oestrous females. Similarly, a male of high quality may be preferred by the females, and will pay a lower grooming price to be favored by them. This prediction has been supported by data coming from chimpanzees. In this species, low-ranking males need to provide more grooming to oestrus females than high-ranking males in order to gain access to females [56]. As in other primate species, grooming also seems to play an important role in sifaka. Norscia et al. [22] found that in the months immediately preceding the mating season, male grooming of females positively correlated with female grooming of males. In the mating period, this correlation disappeared because grooming was exchanged by males for copulations (“grooming for sex” tactic). Therefore, it is not surprising that during the mating season clean-chested males, due to their low testosterone levels and consequent low production of secretions (this paper; [33]), invest much more in the “grooming for sex” tactic with females than stained-chested males do. In contrast, the birth season was characterized by a lack of difference in the grooming received by females from the two morphs of males. The “grooming for sex” tactic adopted by clean-chested males during the mating season is not completely unsuccessful; in fact, half of the clean-chested males under study did copulate with females, even though their copulation frequency was significantly lower than that of stained-chested males (Figure 2). The observation that copulation frequency is higher in stained-chested males (usually dominant in their social group; [26]) than in clean-chested males is consistent with the paternity test results presented by Kappeler and Schäffler's [57], showing that sifaka dominant males can sire up to 90% of infants.

In conclusion, since the badge depends on testosterone, scent-marking, and dominance, it can represent an “overview” of males' physical state. To demonstrate the function of a potential communicative signal the experimental approach is generally required, unfortunately such approach is not feasible with this species.

Our findings that females copulate more with males showing chest stain suggest that this cue is used by females to choose mates. The choice pattern could also result from correlated expression of the stained chest with other cues that the females directly target. The clues conveyed by the badge may be used as an additional piece of information to assess the potential quality of stranger males, possessing cues that cannot be timely accessed by females.

The presence of the multiple mating tactics, “marking for sex” (stained-chested males) and “grooming for sex” (an alternative, but not completely functional, tactic used by clean-chested males) may be a means by which sifaka population buffers the inbreeding phenomenon in the small, isolated fragment of the Berenty forest [58].

## Materials and Methods

### Ethics statement

This study was approved by University of Pisa (Animal Care and Use board). Since the study was purely observational the committee waived the need for a permit. The study was conducted with no manipulation of animals. The study was carried out in the private Reserve of Berenty (South Madagascar) and De Heaulme family (the owner) permitted us to observe animals.

### Study species and site

We conducted this study in the secondary forest of Ankoba, part of the 140-ha Berenty forest fragment (South Madagascar; S 24.99°; E 46.29°; for an extensive description see [59] on *Propithecus verreauxi* (Verreaux' sifaka). At Berenty, sifaka groups range from 1 to 10 individuals, according to a complete census conducted in November-December 2006 [60]. They inhabit riverine and dry forests of south and southwest Madagascar [61]. Females usually experience a single oestrus period (2–3 days) per year and both sexes can mate with multiple partners in their own and neighbouring groups, especially when a single group offers suboptimal mating opportunities [52]. In particular, males can start roaming and visiting other groups in search of oestrus females [21]. The short oestrus period and the fact that mating can be tightly synchronized within a population make copulations very difficult to detect and observe [23], [26]. Moreover, at Berenty, cyclones and heavy raining followed by river flooding normally prevent data collection in the period January-February, coinciding with sifaka's mating period. In 2007, for the first time it was possible to gather data on mating because of a prolonged drought involving South Madagascar. In the end, we gathered the highest sample of mating episodes (57 copulations) ever recorded in sifaka [22]. In May-July 2008, during the birth season, we gathered data on the same groups. This additional sample collection permitted us to compare data on marking behaviour and male-female grooming between the two different seasons (mating 2007-birth 2008).

### Observational data and operational definitions

The study was conducted on adults of two sifaka groups in two different periods (mating season: 11 adult males, 6 adult females; birth season: 6 adult males, 6 adult females). Within the out-group males observed in the mating period, 2 were stained-chested and 3 were clean-chested. Animals were followed from dawn to dusk by *focal* (collection of grooming data) (mating season: 501 hours, birth season: 368 hours) and *all occurrences animal sampling* (collection of olfactory activity and copulation data) (mating season: 221 hours, birth season: 258 hours). During the mating season the authors and a field assistant collected data with daily observations of about 11 h/day. During the birth season, due to the reduced day length, the observations decreased to about 9 h/day. As typical of the sifaka the individuals of the group usually moved, rested, and foraged cohesively. However, the group could split during the mating days: in this case, the observers separated to follow the two different subgroups. We individually identified the animals according to their external features (scars, fur patches, fur color, [62]).

To distinguish stained- and clean-chested males we used the descriptions given by Lewis and van Schaik [26]. We photographed males' chest at a maximum distance of 2 m. Males with a brown, greasy spot on the chest were labeled as “stained”, whereas males with a white, clean chest were identified as “clean”. The animals with intermediate color were two out-group males (one per group) which spent in our study groups only few hours in a day. For this reason we decided to exclude them from the analysis.

Brockman [21], who observed sifaka mating in a different study site (Beza-Mahafaly; Southeastern Madagascar), provided the operational definitions used during this study (Table 1). We included in the analyses only proper copulations.

### Statistical analyses

Due to the small sample size and deviation from normality (Kolmogorov-Smirnov<0.05) we used randomization procedures ([63], software: Resampling Procedures 1.3 by David C. Howell, freeware). Specifically, randomization tests were employed with a number of 10,000 permutations using resampling procedures. The software provides a t value in the same way as in a standard t test, but calculates a p value as the proportion of randomized datasets that yield an even more extreme outcome. The analyses were conducted at an individual level. All analyses were two-tailed, and the level of significance was set at 5%.
